# Optimizing future well-being with artificial intelligence: self-organizing maps (SOMs) for the identification of islands of emotional stability

**DOI:** 10.18632/aging.204061

**Published:** 2022-06-20

**Authors:** Fedor Galkin, Kirill Kochetov, Michelle Keller, Alex Zhavoronkov, Nancy Etcoff

**Affiliations:** 1 Deep Longevity Limited, Hong Kong; 2 Insilico Medicine, Hong Kong; 3 Buck Institute for Research on Aging, Novato, CA 94945, USA; 4 Department of Psychiatry, Massachusetts General Hospital, Harvard Medical School, Boston, MA 02115, USA

**Keywords:** self-organizing maps, well-being, aging, artificial intelligence, depression

## Abstract

In this article, we present a deep learning model of human psychology that can predict one’s current age and future well-being. We used the model to demonstrate that one’s baseline well-being is not the determining factor of future well-being, as posited by hedonic treadmill theory. Further, we have created a 2D map of human psychotypes and identified the regions that are most vulnerable to depression. This map may be used to provide personalized recommendations for maximizing one’s future well-being.

## INTRODUCTION

### Psychological well-being

With the surge in mental illness, well-being has attracted the attention of many government agencies and research groups worldwide. In 2017 “Our world in Data” estimated that 792 million people live with a mental health disorder, with depression and anxiety disorders leading the list [1]. In 2018 that estimate had risen to one billion globally. The COVID-19 pandemic has only exacerbated the mental health situation, according to several recent reports [2–5]. Growing psychological distress, boredom, and feelings of isolation and loneliness have led to a decrease in well-being that is hampering society’s productivity and ability to endure these challenging times.

Decades of well-being research have identified psychological well-being as tightly linked to physical health, and to optimism and supportive social networks. Well-being can be conceptualized as a positive mood and outlooks or as a sense of flourishing or positive functioning. High well-being is associated with positive health behaviors, lower risk of cardiovascular events, and all-cause mortality [6–9]. Among almost one hundred scales used to measure well-being, the Ryff scale is one the most widely used ones [10, 11]. The six dimensions of well-being defined within this scale are (i) autonomy, (ii) environmental mastery, (iii) personal growth, (iv) positive relations, (v) purpose in life, and (vi) self-acceptance. The original scale comprises 20 questions for each aspect of well-being, although a truncated version with only three questions each is also available [12]. The truncated version of the scale was used in the Midlife in the United States study (MIDUS), which we explored to study personal well-being trajectories [13, 14].

MIDUS contains almost four thousand subjects with known psychological profiles at two time points (MIDUS1 collected at 1995-1996 and MIDUS2 collected in 2004-2006), including their well-being profiles (Table 1). This unique dataset allowed us to explore the age-dependent component of well-being and to address the questions of its stability. The U-shape hypothesis posits that people experience minimal well-being in middle age, a claim that is still being argued actively [15, 16]. The results of studies addressing this phenomenon are highly dependent on the data source and methodology. Some studies have reported that well-being is mostly static within a smaller timeframe [17]. In some cases, no age effects are detected on a lifetime scale [18]. The concept of static well-being is frequently referred to as the “hedonic treadmill,” the idea that even after major events, people return to their baseline level of life satisfaction. This concept, however, is an inaccurate representation of reality, since well-being adaptation has been shown to be avoidable [19, 20]. While research has suggested that happiness levels are durable, withstanding sweeping changes in health and wealth, the extreme picture of the hedonic set point as inevitable for all is in need of revision. While the average person’s happiness may return to baseline, the happiness of many individuals does not [21]. In part, the fluidity of overall life satisfaction can be explained by the multifaceted nature of well-being, and each aspect has a different response rate to life events. We show that there are quite distinct non-constant trajectories for the six well-being parameters.

**Table 1:** The variables of interest studied in this article.

**MIDUS1 variable**	**MIDUS2 variable**	**Description**
A1SPWBR	B1SPWBR1	Positive relations
A1SPWBS	B1SPWBS1	Self-acceptance
A1SPWBA	B1SPWBA1	Autonomy
A1SPWBG	B1SPWBG1	Personal growth
A1SPWBE	B1SPWBE1	Environmental mastery
A1SPWBU	B1SPWBU1	Purpose in life
A1PAGE_M2	B1PAGE_M2	Age
A1PDEPAD	B1PDEPAD	Depressed affect
A1PDEPDX	B1PDEPDX	Depressed affect + anhedonia

### mHealth apps and self-help

Data-driven approaches to mental health are a rapidly developing field of psychology. The widespread use of smartphones has enabled novel ways to monitor behaviors and interact with people in need of guidance. According to recent market research, the mHealth app industry will continue expanding at a 28% annual growth rate, indicating great demand [22]. Compared to face-to-face psychology sessions, the mHealth approach allows for more frequent interactions at a greatly reduced cost. Reviews of this technology suggest that internet-based cognitive behavioral therapy (CBT) reduces the frequency and severity of depressive symptoms [23].

The models reported in this article may be used to develop online CBT tools for self-improvement, initial screening, or therapist-guided interventions.

The online mental health approach, however has serious shortcomings. First of all, mHealth CBTs tend to have double the dropout rate of clinician-based alternatives at 74%, thus demonstrating the necessity of therapist support [24]. In part, high dropout may be caused by insufficient personalization of self-help methods and a lack of professional oversight to achieve the desired change. This may not be an issue in academic studies with smaller samples and well-defined purposes, such as treating insomnia or anxiety [25–27]. However, the variability of psychological problems the general population encounters necessitates an online tool that can adapt to its users and their unique psychological profiles.

Deep learning approaches, such as neural networks and self-organizing maps (SOMs), can carry out initial assessment automatically and serve as recommendation engines to provide personalized daily tips. We expect that user interactions guided by the models described in this article can create better user satisfaction and lead to better retention.

### The AI approach and aging clocks

Over the last decade, deep learning techniques have spread to the point of ubiquity. This blanket term encompasses a wide variety of data-driven algorithmic models used in synthetic data generation, image recognition, signal denoising, decision making, chatbots, recommendation engines, but most importantly to the scope of this article — in clinical settings.

Deep learning algorithms excel in tasks that require discerning non-linear dependencies, provided a sufficiently large training set is provided and the task is well formalized. In application to life sciences, deep learning has been demonstrated to successfully solve the tasks of pharmaceutical lead selection and drug design, clinical trial design, as well as diagnostic imaging and electrodiagnosis [28–32]. AI systems have a virtually unlimited bandwidth, compared to human professionals, and can deliver their reports in a matter of seconds, thus enabling more affordable and scalable healthcare [33].

The rapid development of deep learning has eventually reached the field of biogerontology in which neural networks were used to create digital models of aging — aging clocks. Such models allow measuring the intensity of the aging-related processes in the human body and were introduced in a seminal paper by Steve Horvath in 2013 [34]. The original publication featured a linear model that predicted human chronological age based on their DNA methylation profile. Since then, aging clocks have been trained on practically any type of bio-relevant information, such as facial photos, transcriptomes, clinical blood tests and more [35–43]. The algorithms behind aging clocks have also become more sophisticated and now modern instances feature deep learning solutions as well as models trained to predict frailty or mortality risk directly [44–48].

Although the use of aging clocks is currently limited to research settings, this technology carries the promise of serving as the foundation for a disruptive model of global healthcare, longevity medicine [49–57]. The pace of aging detected with aging clocks has been associated with higher all-cause mortality risk, frailty, and the development of aging-related diseases [58–60].

Many factors of everyday life have been studied in the context of their effect on the pace of aging, including psychological factors such as stress. Childhood stress, traumatic experiences, intense competition, or experiencing violence have been shown to increase the biological age identified with epigenetic aging clocks [61–64]. The supposed mechanism linking stress and the pace of aging involves aberrant glucocorticoid signaling disrupting the DNA methylation patterns in key aging loci [65, 66]. Other studies also outline subjective age as major factor in overall well-being and all-cause mortality [67].

**Figure 1:**
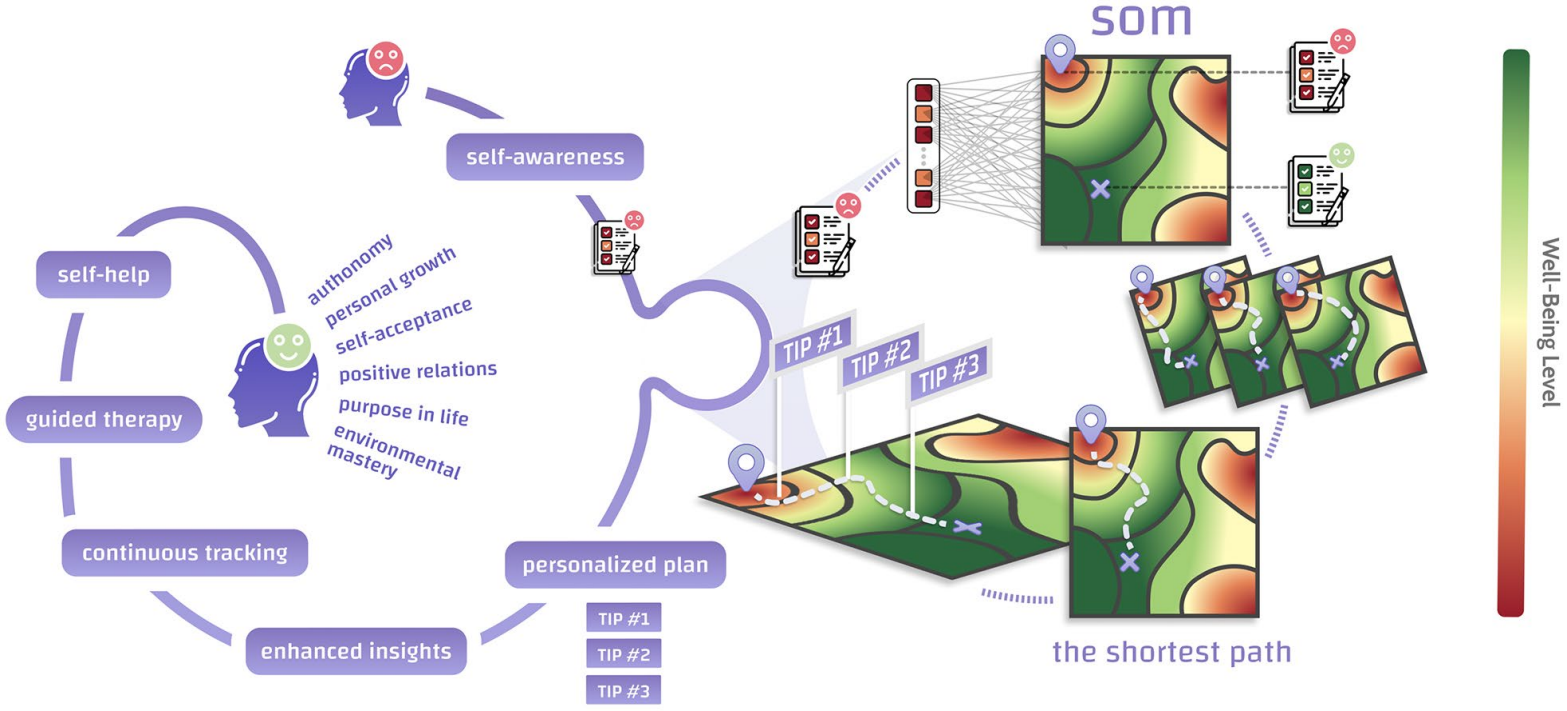
**We have created the backbone of an AI-assisted recommendation engine to improve current and future psychological well-being based on self-organizing maps (SOMs).** A person seeking self-improvement fills in a psychological test and is placed on a 2D representation of the multidimensional space containing all possible psychotypes. The map consists of regions associated with high (green) and low (red) well-being, which may be considered “mountains” and “pits”. Distance metrics defined within a SOM allow finding the shortest path between a person’s starting point and the point that maximizes their well-being. One’s journey across the SOM may be interpreted as a chain of incremental changes leading to higher well-being. The SOM offers non-trivial, personalized paths towards improved well-being that can be followed and tracked within a self-help app or during therapy sessions.

In this article, we continue to explore human psychology and its lifetime evolution. The longitudinal setting of the MIDUS dataset enabled us to create a deep learning predictor of future well-being and psychological age. Furthermore, we used SOMs to visualize human psychotypes and derive the paths of incremental lifestyle changes that should lead to an increase in current and future well-being (Figure 1). The deep learning approach allowed us to capture the complex, non-linear properties of the human psyche. The general approach described here can easily be implemented as the core of an mHealth application to help people improve their mental resilience.

## RESULTS

### Deep learning predictors of age and future well-being

We employed Boruta feature selection to identify all MIDUS features that may be associated with future well-being to obtain a list of 32 variables (Supplementary Figure 1A and Table 2), which were used to train an age predictor and a well-being predictor. Since the selected features are not, by design, associated with age, the final model only marginally outperformed the median age assignment, displaying mean absolute error (MAE) of 9.06 years in the test set (Supplementary Table 1). The predicted psychological age may be further corrected for chronological age to make it representative of the typical aging rate in each particular age group. This procedure boosted the accuracy of the model to an MAE of 4.30 years, nevertheless completely transforming the meaning of its output. Thus, the prediction is no longer a chronological age estimate but a metric of psychological similarity to age peers.

**Table 2:** Features used to train the SOM and the predictors.

**Selected features**	**Description**
A1PD1	Satisfied with life at present
A1SF1C	Some wander aimlessly, but not me
A1SF1D	Demands of everyday life often get me down
A1SF1F	Maintaining close relationships difficult
A1SF1I	Good managing daily responsibilities
A1SF1K	Life process of learning/changing/growth
A1SF1L	Experience challenge how think important
A1SF1M	Others describe me as giving/share time
A1SF1U	Do just about anything I set my mind to
A1SF1X	When really want something, find way
A1SF1Y	Many things interfere with what I want do
A1SF1Z	Whether I get what want is in own hands
A1SF3B	Do what can to change for better
A1SF3P	Know what I want out of life
A1SF3Q	I live one day at a time
A1SF3T	Helpful to set goals for near future
A1SF3W	No use in thinking about past because nothing can be done
A1SF4A	Outgoing describes you how well
A1SF4D	Organized describes you how well
A1SF4Y	Broad minded describes you how well
A1SF4Z	Sympathetic describes you how well
A1SK10A	Give spouse/partner emotional support (hours/month)
A1SK17A	World is too complex for me
A1SK17F	Feel close to others in community
A1SK17G	Daily activities not worthwhile for community
A1SK17J	People do not care about others problems
A1SK17M	Society not improving for people like me
A1SK17N	Believe people are kind
A1SK7I	Serve on a jury if called
A1SK7Q	Volunteer for social causes
A1SM13	Rely on friends for help with problem
A1SM5	Open up to family about worries

Future well-being predictions were not adjusted, and the accuracy of predictions ranged between mean absolute percentage error (MAPE) of 15.81% and 22.53% for different aspects of well-being in 10 years (Supplementary Table 1). The most accurate predictions were obtained for personal growth, while estimates of future self-acceptance were the least accurate.

### Relative effect of deep learning features on well-being

To illustrate the reliability of the generated future well-being estimates, we performed a relative importance analysis using an elastic net (EN) method. Actual well-being features in the follow-up MIDUS2 wave were regressed as a linear combination of sex, age, the corresponding predicted well-being variable, and all six MIDUS1 well-being features (Supplementary Table 2). In all such models, model-derived estimates showed the largest coefficients, thus identifying them as more important determinants of future well-being than current well-being.

### SOM-based recommendations for improving well-being

The 32 selected variables form a space of psychotypes that is representative of the general US population. To visualize and navigate these psychotypes, we used SOMs—a method of dimensionality reduction that may be interpreted as a 2D projection that preserves the distances between samples.

The SOM we obtained contains 625 cells organized in a 25 × 25 grid. Each psychological profile matches only one cell, while each cell may have multiple matching profiles. By design, SOMs ensure that subjects with similar psychotypes occupy the same cell. Conversely, more dissimilar psychotypes will be farther apart on an SOM.

SOMs provide a way to explore metadata and assess whether people with similar psychotypes (the same SOM region) share any properties not included in the initial 32 variables. Within the scope of this article, we have focused on the well-being structure of SOMs. Originally, we intended to describe all six well-being parameters with SOMs. However, for the sake of brevity, we have used a proxy of well-being in the main text, and the SOM distributions for all six parameters are available in the Supplementary Materials.

Our results showed that the odds of depression for people mapped to the same SOM cell were significantly negatively correlated with all current and four out of six future well-being parameters (Supplementary Table 3). Thus, we established depression odds as a reliable proxy for well-being.

**Figure 2:**
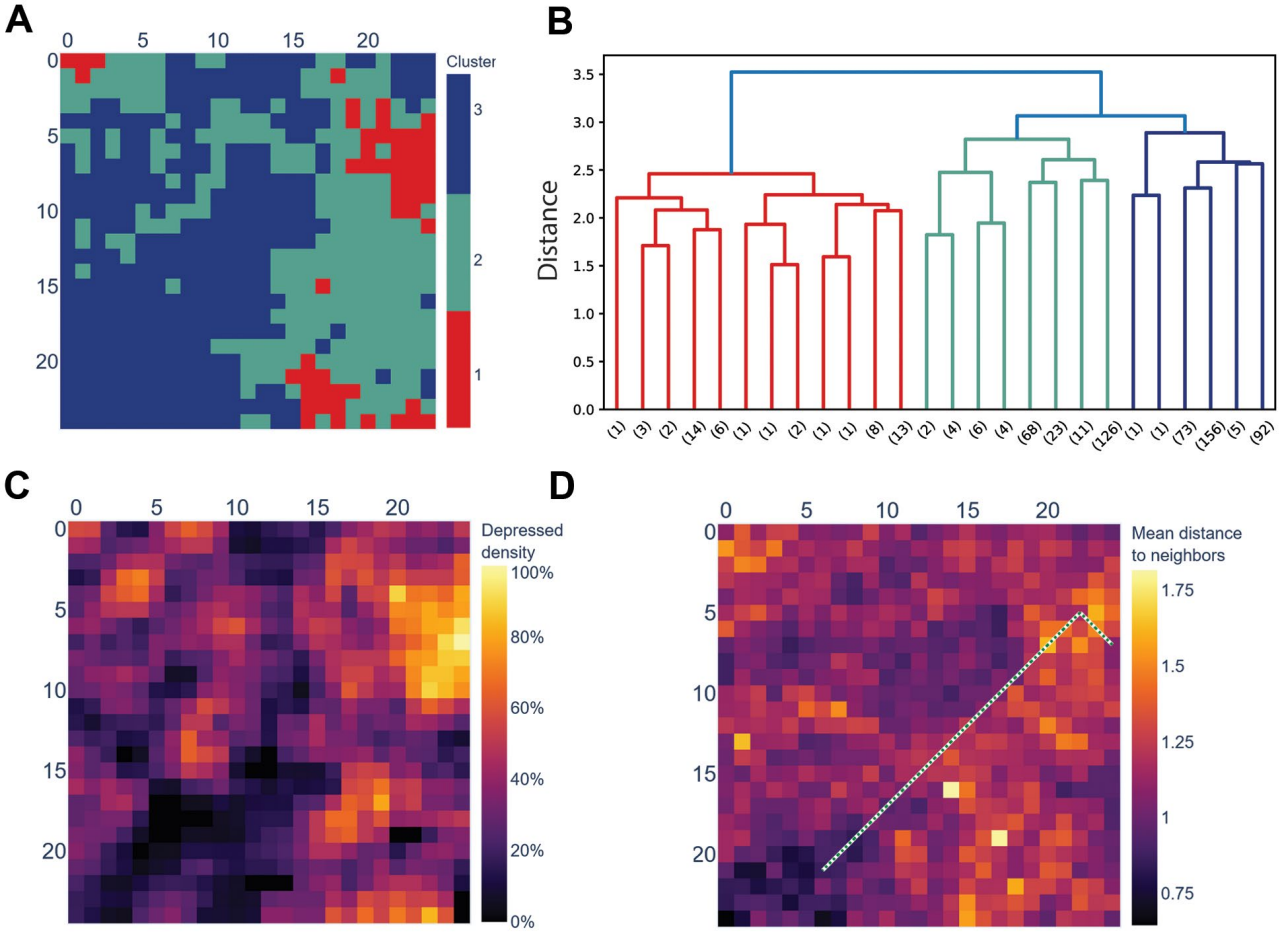
**The SOM trained on a cohort of non-depressed people separates the depressed and the non-depressed.** (**A**) Hierarchical clustering of SOM’s nodes identified three clusters; (**B**) Dendrogram of the clusters displayed in section A. Distance is Euclidean distance between clusters (complete linkage); numbers in brackets mark the number of leaves below the pruned branches; (**C**) Cluster-1 displayed in section A coincides with SOM’s cells to which more depressed, rather than non-depressed, respondents from the test cohort (N = 1173) are mapped. NA (dark green) marks the cells to which no respondents from the test cohort were mapped; (**D**) SOM colored by the average distance between a cell and its neighbors (U-matrix). The green dotted line is the shortest path between the cell with the most depressed respondents (top-right) and with the most non-depressed respondents (bottom-left).

Although the SOM was trained on a cohort of non-depressed people, it contained three distinct clusters with significantly different affinity for depressed people from the test set (Figure 2, Table 3 and Supplementary Figure 1B). Respondents mapped to the smallest Cluster-1 had 2.15 odds of being depressed, while in the two other clusters, non-depressed people outnumbered the depressed. Similarly, future well-being in all six aspects was significantly different among the three clusters, with Cluster-1 showing the lowest future well-being scores.

**Table 3:** Overview of the three clusters identified within the SOM.

	**Cluster-1**	**Cluster-2**	**Cluster-3**	**Total**
Number of cells	53	244	328	625
Number of depressed people in the test set	71	179	144	394
Number of non-depressed people in the test set	33	261	485	779
Depression odds	2.15	0.69	0.30	0.51
% male	31.73	40.00	41.34	39.98

Further, we examined the features that made Cluster-1 different from the other two clusters. To compare people from different clusters, rather than the depressed versus the non-depressed, we considered only the non-depressed 779 respondents from the test cohort in this section. We observed people with certain mindset features were more likely to be categorized into the depression-prone Cluster-1 (Supplementary Figures 2–4). For example, people in Cluster-1 were less likely to seek personal growth (A1SF3B), had a more vague understanding of life (A1SF3P), were more narrow-minded (A1SF4Y), and were less outgoing (A1SF4A). Other variables in cluster-1 and Cluster-2 that differed significantly (p <0.001) include: life satisfaction (A1PD1), being organized (A1SF4D), and being open with friends (A1SM13) and family (A1SM5) about one’s problems.

Some differences between clusters showed that people in Cluster-1 felt lost and out of control (A1SF1C, A1SF1U, A1SF1X, and A1SF1Z) and were less likely to use problem-focused coping mechanisms (A1SF1K, A1SF1L, A1SF1X, and A1SF1Z). See Supplementary Figures 2–4.

**Figure 3:**
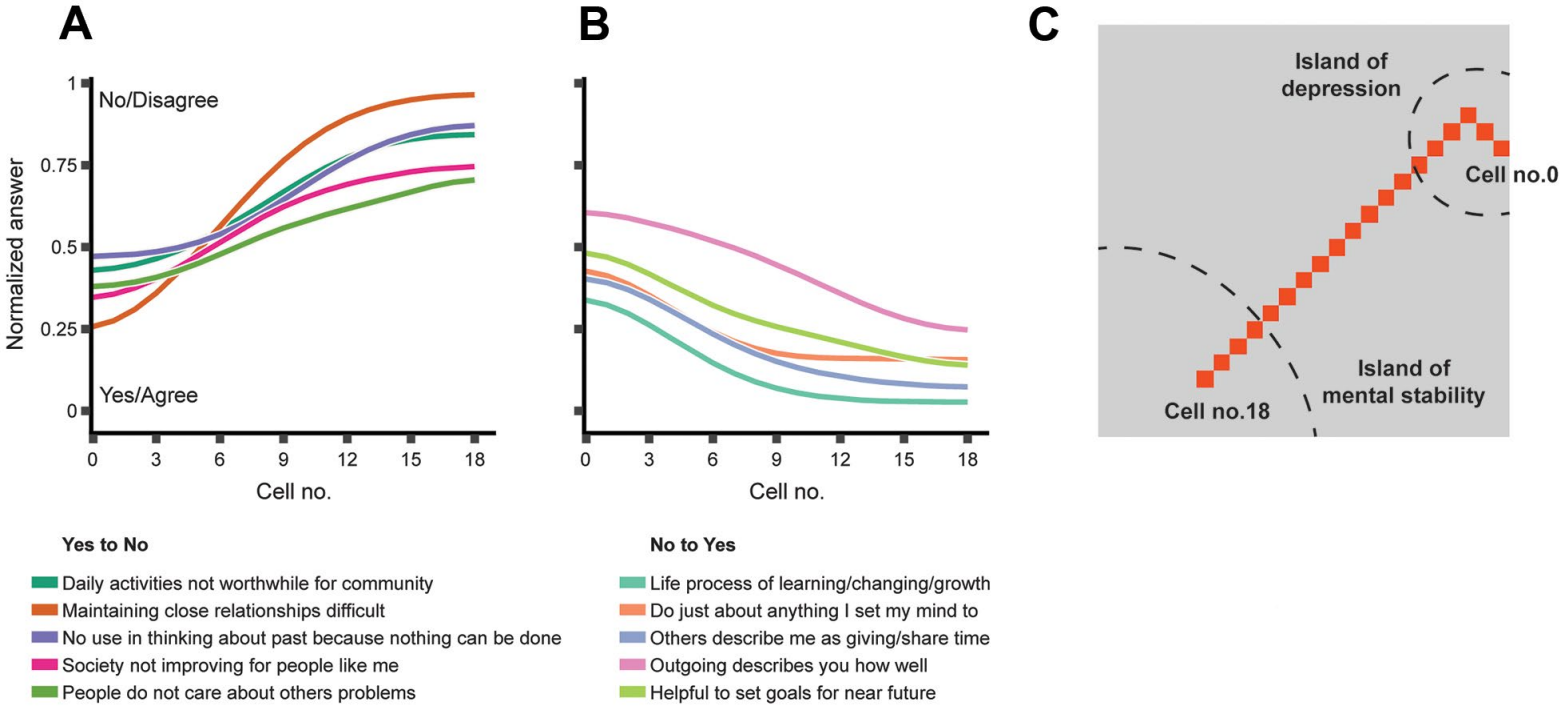
**The features that change the most along the path between the respondents mapped to the “island of depression” and those mapped to the “island of mental stability.”** (**A**) Top-five attitudes that are generally not shared by mentally stable people but are prevalent among depressed people; (**B**) Top-five attitudes that are prevalent in mentally stable people but generally not shared by depressed people; (**C**) The shortest path connecting the SOM cell with the highest prevalence of depressed people (cell-0) to the cell with the highest prevalence of non-depressed people (cell-18). The curves in Panel A and Panel B represent the feature vectors stored in the SOM cells on the path from cell-0 to cell-18. The path displayed in Panel C is also marked in Figure 1D.

The geometric nature of an SOM allowed us to treat it as an actual map to mark paths connecting regions of interest. In this particular case, we chose to mark the path between the depression hotspot in Cluster-1 and the most depression-resilient cell in Cluster-3 (Figure 2D). This path might be interpreted as a sequence of gradual changes that a depressed individual should go through to overcome their condition (Figure 3).

To complete the transition, a respondent would need to change certain attitudes and behaviors. Some of the identified features were trivial and difficult to manipulate; for example, overall life satisfaction (A1PD1) increased toward Cluster-3. Other features offered more convenient therapy targets, such as increasing community involvement (A1SK17F) or openness with family (A1SM5).

A similar approach may be used for a person projected onto any other cell of the SOM to derive primary CBT goals and quantify their effects on well-being or depression symptoms in advance.

## DISCUSSION

In this study, we explored the MIDUS dataset to describe the age-dependent component of well-being and to develop a framework for helping people improve their long-term life satisfaction.

To address the first part of our research goal, we pre-selected 32 psychological variables associated with well-being and created an age and a future well-being predictor. The age predictor, quite expectedly, showed poor performance at MAE = 9.06 years, since the features it was built on were not chosen to be representative of age. On the contrary, as part of our feature selection process, we deliberately removed any variables that might have leaked age. For example, the number of children, income, and the number of comorbidities are expected to be good predictors of age. However, they provide few actionable items and do not represent one’s psychological state, and any analysis of these features in the context of aging will offer only trivial results.

The 9.1-year accuracy is marginally better than the baseline but still falls short of the 6.7-year MAE, another similar aging clock, PsychoAge, demonstrated in MIDUS [68]. Thus, we chose not to use the new age predictor to describe the connection between age and well-being.

Well-being as a function of age can be easily visualized to verify the U-shaped hypothesis (Supplementary Figure 5). Since the well-being score distribution in MIDUS is heavily skewed to the right, the mean or median well-being levels were not representative of its age trajectory. Instead, we used the portion of people above 90% of the maximal score as a function of age. Within this definition, the predicted and the real age trends closely resembled each other (MAPE <5.4%). However, neither one of them have been U-shaped, as described elsewhere [16]. This may be due to the inherent differences in well-being scales, or due to MIDUS using a truncated version of the Ryff scale. In this study, certain well-being parameters were observed to trend upward with age (positive relations, environmental mastery, and autonomy), whereas others trended downward (purpose in life and personal growth). In the context of psychological aging, this outcome indicates that there is no “optimal age” at which people reach the apex of well-being. Similarly, there is no “age of unhappiness” at which people experience all-round low well-being. Since different aspects of well-being do not uniformly depend on one’s psychological age, using the AI-derived metrics of age in practice requires caution. Psychological rejuvenation as a target should be considered within the context of personal priorities and be adjusted according to which well-being aspects a person considers most important.

As we have observed, the “positive relations” aspect of well-being is higher in older people while more exploratory, future-oriented aspects such as “personal growth” and “purpose in life” are higher in the youth (Supplementary Figure 5). This age-related “conversion” of well-being is well described within the socio-emotional selectivity theory by Laura Carstensen [69]. The theory posits that as people grow older they “shift from preparatory to consumptive goals”. The choices dedicated to exploration or long-term benefits, such as building a career, self-improvement, or a quest for the meaning of life, are ranked higher than the choices promising emotional comfort by younger people. As people grow older and their time horizon dwindles, they start to value positive emotions more, especially when choosing social partners. Interestingly, a series of studies has shown that a similar effect is observed in people presented with the necessity to relocate or people primed with death reflection [70–72]. This suggests that psychological age, as defined in this article, should be malleable and represents one’s longevity optimism as well as one’s chronological age. Interestingly, new research suggests that in individuals 85 to 90 years old, all measures of optimism were associated with improved 5 year survival [73].

To verify whether the hedonic adaptation hypothesis holds true, we performed a relative importance analysis with EN. In this experiment, we compared whether future well-being estimates (provided by our predictor) or current well-being were a better predictor of actual future well-being. For all six well-being parameters, the deep-learning-derived predictions were significantly closer to actual future well-being than current well-being levels (Supplementary Table 2). If the hedonic adaptation hypothesis was true, the opposite should have been observed, with future well-being being influenced more by baseline well-being levels.

Lastly, we set out to develop an SOM-based recommendation engine that provides personalized self-improvement goals. SOMs are a type of unsupervised learning model that shares technological similarities to neural networks. An untrained SOM stores a vector of length *N* in each of its cells, also called neurons. Then, it is trained by presenting it with data vectors of length *N*, each one slightly adjusting the values in the neurons according to an activation function so that the topological structure of the N-dimensional dataset is preserved. In the end, a 2D representation of a multidimensional space is obtained. The basic operation for an SOM is matching or finding a neuron that stores a vector that is the most similar to a newly presented data instance. Based on the position of the best-matching unit (BMU), useful insights may be derived for the new instance.

SOMs have already been used to describe mental health in humans. For example, an SOM classifier has been trained to determine mental disorders in based on patients’ transcribed speech [74]. SOMs do not require sample labels to be trained which greatly facilitates data collection and preparation stages of a research project. The cited model has reached high (97%) accuracy while predicting the type of a mental diseases. SOMs have also been employed to detect psychological stress based on biometric data from wearables and phone usage [75]. These models, however, are not based on easily interpretable and actionable features, and thus, are not fit to serve as an mHealth application or a therapy tool.

In this study, an SOM was trained on a collection of non-depressed people and validated on a smaller set of both depressed and non-depressed MIDUS participants. When presented with a new sample, the SOM can return future well-being levels and depression risk to it based on the metadata of any known samples mapped to the same BMU. Most importantly, the SOM can be used for initial screening and highlighting mindset aspects that should first be addressed to optimize a person’s long-term well-being.

Applying hierarchical clustering to the SOM yielded three clusters, aggregating significantly different people in terms of well-being and depression status. Interestingly, the clusters also had different sex distributions, with the depression-prone Cluster-1 being predominantly female, which is in line with existing studies of depression [76].

Features contributing to one’s placement in Cluster-1 may be considered primary therapy targets. For example, feeling burdened by close relationships (A1SF1F) is the feature that differentiates depression-susceptible and mentally stable people the most, according to our model (Figure 3 and Supplementary Figure 3). Thus, a therapy focused primarily on building deep, valuable relationships might be more effective in general compared to a therapy focused on goal-setting (A1SF3T) or social involvement (A1SF4A, Figure 3, and Supplementary Figure 2). The demonstrated path to mental stability would only apply to some people, while the primary therapy targets may be different for individuals who belong to other BMUs. Moreover, different well-being densities across the SOM suggest that the path may be adjusted based on the priorities of a particular person. It is expected that the efficiency of certain therapeutic techniques may also be influenced by factors not present in our model. For example, physical attractiveness is positively correlated with psychological well-being and social warmth [77, 78]. A multitude of lifestyles, habits, and other unaccounted factors can affect how fast a person progresses along an SOM-defined path. Thus, a follow-up study is required to estimate the effect such factors have on one’s placement in the SOM.

**Figure 4:**
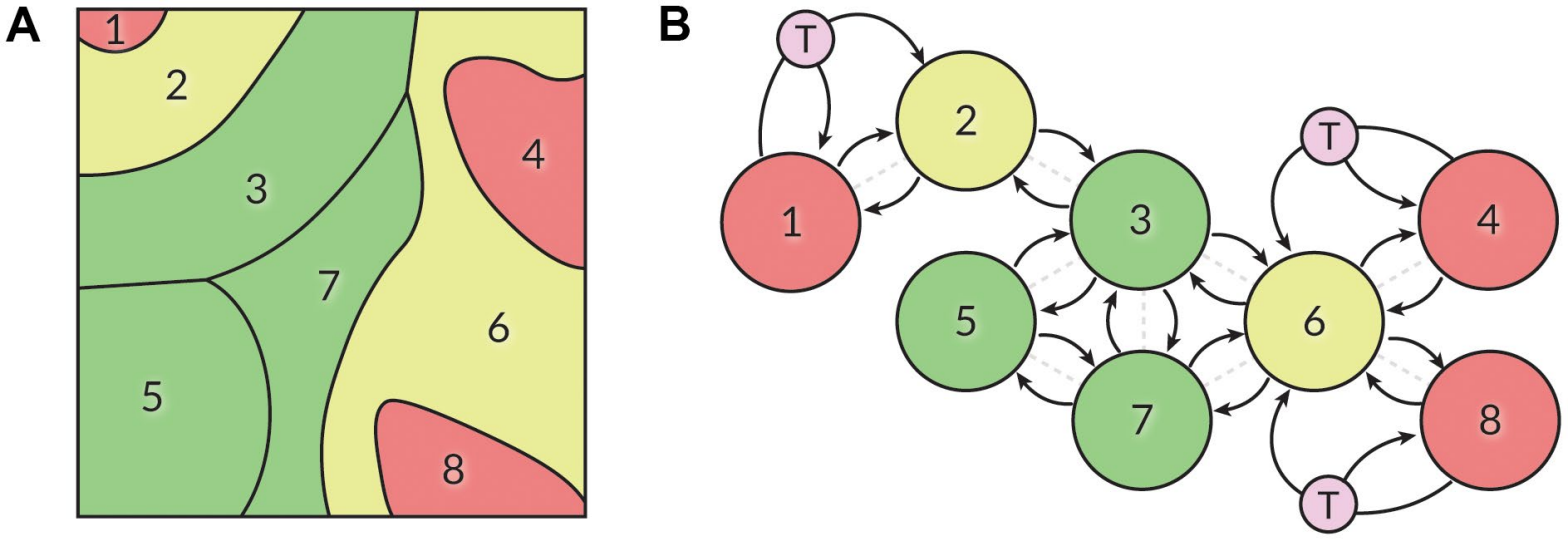
(**A**) The SOM displayed in Figure 1A may be partitioned into eight sections based on the well-being and propensity for depression of people contained within them. Green – highest well-being, yellow –intermediary state, red – low well-being. (**B**) We hypothesize that a person’s position in the SOM is not constant and may be described with a Markov process. People may freely roam within the defined sectors, while transitioning between sectors is equivalent to state transitions in a Markov chain. Actions such as therapy (T) may affect transition probabilities, transforming the model into a Markov decision process.

We also propose that these psychological trajectories may be modeled as a Markov process containing clusters of well-connected states and “bridge” states connecting different clusters (Figure 4). Some attempts to model major depressive disorder as a Markov process have been made before [79, 80]. The cited works explored the cases of two-state and four-state chains, as well as constant or periodic transition probabilities. In some works, depression is conceptualized as a partially-observable Markov decision process [81]. We believe that our findings can serve as a starting point for a practically applicable mathematical model to unite our understanding of well-being, aging, and mental health. Such modeling would require continuous data collection to observe the fluidity of in-model psychotypes and approximate state transition probabilities.

## Conclusion

In this article, we presented a deep learning model based on a psychological questionnaire that can be used to estimate one’s psychological age, future well-being, and risk of depression. Based on the same questionnaire, we described an SOM that contained three distinct clusters characterized by different well-being levels and propensity for depression. The SOM can be employed for the purposes of cognitive behavioral therapy and online mental health approaches. Such a tool when implemented may be used as an initial and follow-up assessment tool in face-to-face therapy, or as a standalone consumer application to improve well-being. We also hypothesize that the transitions between the three clusters of psychological profiles may be modeled as a Markov chain for future practical applications.

## MATERIALS AND METHODS

### Data

The samples used in this article were obtained from MIDUS1 (1995-1996) and MIDUS2 (2004-2006) datasets. Their participants provided information over phone interviews and self-administered questionnaires.

Only the participants present in both MIDUS iterations were included in the study. The target variables used to train models and detect aging-related psychological trends are listed in Table 1. Subjects with missing A1PDEPDX (depression or anhedonia) values were excluded.

The six MIDUS well-being variables are based on the Ryff scale [11]. While the original Ryff scale allocates 20 questions to each aspect of psychological well-being, the MIDUS version of the well-being scale uses only three questions. These questions did not change between MIDUS1 and MIDUS2, apart from a slight change in one of the autonomy questions.

The 3891 participants were separated into training (N = 2718) and test (N = 1173) sets. The test set contained 779 non-depressed individuals and 394 marked as depressed in MIDUS1. No depressed individuals were included in the training set.

Any Supplementary Files related to this study have been deposited to a public Open Science Framework repository [82].

### Feature selection

To create the psychological aging clock and the six future well-being predictors, a multi-stage feature selection procedure was employed (Supplementary Figure 1A).

During the first stage, all 2097 MIDUS1 variables were considered. All features relevant to either one of the MIDUS2 well-being features were selected using Boruta v0.3 for Python 3.9 [83]. The union of all relevant features contained 135 variables.

During the second stage, we filtered all the selected features. All non-modifiable features were excluded, and all composite features were deconvoluted into their constituent questions.

For example, A1SEFA (“Paternal affection”) was excluded because it represents historical information that cannot be changed. A1SPIWOR (“Perceived inequality in work”) was excluded because modifying this feature for a respondent might mean quitting their job to find another—an action associated with the financial risk most people are unwilling to take. A1SKINPO (“Support from family”) is a score calculated from four other questions, so it was replaced with A1SM2, A1SM3, A1SM4, and A1SM5.

After filtering the non-modifiable features, 101 variables remained. Boruta was applied once again to determine the constituents of the relevant composite features. All features from Boruta’s output were subjected to a collinearity test, and all features with a variance inflation factor above 10 were excluded. The variance inflation factor was calculated with the statsmodels v1.16.0 package for Python 3.9.

Eventually, 32 variables remained, as listed in Table 2. Among these, only seven were from the MIDUS1 well-being survey (out of 18). The flow of our feature selection procedure is presented in Supplementary File 1.

### Model training

We trained two separate regression models, one for age prediction and one for future well-being prediction. The age model returned only one prediction as output, but the well-being model returned all six parameters simultaneously and was trained in a multilabel manner. We used the 32 features remaining after our feature selection for both predictors.

Feed-forward neural networks with more than three hidden layers were used as predictors with Python 3.9 implementation, using the Keras library (https://keras.io/) with TensorFlow backend (https://www.tensorflow.org) to build and train neural networks. We used the grid search optimization technique over the space of model parameters to find the best performing network architecture separately for both prediction tasks. The MAE loss function was used as an objective for deep neural networks.

The best performing model for age prediction had 4 hidden layers with 256 neurons each, Leaky ReLU activation function [84] after each layer, dropout [85] with 25% probability, and L2 with coefficient 1e-6 after each layer for the purposes of regularization.

The best performing model for future well-being prediction had 4 hidden layers with 400 neurons each, Leaky ReLU activation function after each layer, dropout with 25% probability, and L2 with coefficient 1e-7 after each layer for the purposes of regularization.

Neural networks were trained using five-fold cross-validation to compensate for overfitting and to obtain more robust performance metrics.

### Relative importance analysis

To compare whether the current well-being or well-being predicted by MindAge2 was a better determinant of future (MIDUS2) well-being, we used the elastic net (EN) method and treated the variable coefficients as a measure of importance.

EN models were trained to predict either one of the six MIDUS2 well-being features and contained age and sex variables, the corresponding predicted future well-being, and all six MIDUS1 well-being features.

EN was implemented using sklearn v.0.24.2 for Python 3.9. To find the optimal coefficients, a grid search was performed for the L1 ratio (from 0 to 1 with 0.01 step), and penalty weight (1E-5, 1E-4, 1E-3, 1E-2, 1E-1, 0.0, 1.0, 10, 100).

### Model statistics

The metrics used to measure the performance of the models in this article are mean absolute error (MAE), mean absolute percentage error (MAPE), and Person’s r. These metrics are defined as follows:


$$\begin{array}{l} MAE=\frac{1}{N}\sum_{i=1}^{N}|Age_{true,\;i}-Age_{predicted,\;i}|,\;\\ \text{where}\;N\;\text{is}\;\text{the}\;\text{total}\;\text{number}\;\text{of}\;\text{samples} \end{array}$$

$$\begin{array}{l} MAPE=\frac{100}{N}\sum_{i=1}^{N}\frac{1}{Age_{true,\;i}}|Age_{true,\;i}-Age_{predicted,\;i}|,\\ \text{where}\;N\;\text{is}\;\text{the}\;\text{total}\;\text{number}\;\text{of}\;\text{samples} \end{array}$$

### Self-organizing maps (SOMs)

We used the SOM approach to map our feature set into a 2D space to visualize the data and provide recommendations through SOM paths [86].

We found the best-performing SOM in terms of quantization error on the presented data using a grid search optimization technique over the space of SOM parameters. Quantization error was the main measurement used to assess SOM quality; it is an average difference between coordinates of SOM-mapped input samples and their corresponding BMUs. An SOM was trained using 32 features remaining after feature selection. We used the MiniSom library for Python 3.9 to train the SOM.

We found that the following parameters performed better for our task: sigma equal to 1.8, learning rate equal to 0.4, Gaussian neighborhood function, Euclidian activation distance, and an SOM size equal to 25 × 25.

The Sigma parameter defines the radius around an updated neuron, in which neighbors should also be updated. The neighborhood function is used to update neighbor neurons and determine the rate of change around the winner neuron. Activation distance is the distance used to activate the map or as a main distance function.

The SOM was trained until convergence, when the quantization error stopped decreasing.

We used the Bellman–Ford algorithm to find the weighted shortest path between two points on the SOM; distances between neurons were used as weights. Any other graph-based shortest path algorithm can be used, for example, the Dijkstra algorithm.

## Supplementary Material

Supplementary Figures

Supplementary Tables

Supplementary File 1

## References

[1] Dattani S, Ritchie H, Roser M. Mental Health. Our World in Data. 2021. https://ourworldindata.org/mental-health

[2] Nochaiwong S, Ruengorn C, Thavorn K, Hutton B, Awiphan R, Phosuya C, Ruanta Y, Wongpakaran N, Wongpakaran T. Global prevalence of mental health issues among the general population during the coronavirus disease-2019 pandemic: a systematic review and meta-analysis. Sci Rep. 2021; 11:10173. 10.1038/s41598-021-89700-8 33986414 PMC8119461

[3] Wu T, Jia X, Shi H, Niu J, Yin X, Xie J, Wang X. Prevalence of mental health problems during the COVID-19 pandemic: A systematic review and meta-analysis. J Affect Disord. 2021; 281:91–8. 10.1016/j.jad.2020.11.117 33310451 PMC7710473

[4] McPherson KE, McAloney-Kocaman K, McGlinchey E, Faeth P, Armour C. Longitudinal analysis of the UK COVID-19 Psychological Wellbeing Study: Trajectories of anxiety, depression and COVID-19-related stress symptomology. Psychiatry Res. 2021; 304:114138. 10.1016/j.psychres.2021.114138 34388511 PMC8424320

[5] Choi I, Kim JH, Kim N, Choi E, Choi J, Suk HW, Na J. How COVID-19 affected mental well-being: An 11- week trajectories of daily well-being of Koreans amidst COVID-19 by age, gender and region. PLoS One. 2021; 16:e0250252. 10.1371/journal.pone.0250252 33891642 PMC8064534

[6] Boehm JK, Kubzansky LD. The heart’s content: the association between positive psychological well-being and cardiovascular health. Psychol Bull. 2012; 138:655–91. 10.1037/a0027448 22506752

[7] Saadeh M, Welmer AK, Dekhtyar S, Fratiglioni L, Calderón-Larrañaga A. The Role of Psychological and Social Well-being on Physical Function Trajectories in Older Adults. J Gerontol A Biol Sci Med Sci. 2020; 75:1579–85. 10.1093/gerona/glaa114 32384140 PMC7357580

[8] Trudel-Fitzgerald C, Millstein RA, von Hippel C, Howe CJ, Tomasso LP, Wagner GR, VanderWeele TJ. Psychological well-being as part of the public health debate? Insight into dimensions, interventions, and policy. BMC Public Health. 2019; 19:1712. 10.1186/s12889-019-8029-x 31856772 PMC6923969

[9] Ryff CD. Psychological well-being revisited: advances in the science and practice of eudaimonia. Psychother Psychosom. 2014; 83:10–28. 10.1159/000353263 24281296 PMC4241300

[10] Linton MJ, Dieppe P, Medina-Lara A. Review of 99 self-report measures for assessing well-being in adults: exploring dimensions of well-being and developments over time. BMJ Open. 2016; 6:e010641. 10.1136/bmjopen-2015-010641 27388349 PMC4947747

[11] Ryff CD. Happiness is everything, or is it? Explorations on the meaning of psychological well-being. J Pers Soc Psychol. 1989; 57:1069–81. 10.1037/0022-3514.57.6.1069

[12] Ryff CD, Keyes CL. The structure of psychological well-being revisited. J Pers Soc Psychol. 1995; 69:719–27. 10.1037//0022-3514.69.4.719 7473027

[13] Brim OG, Baltes PB, Bumpass LL, Cleary PD, Featherman DL, Hazzard WR, Kessler RC, Lachman ME, Markus HR, Marmot MG, Rossi AS, Ryff CD, Shweder RA. Midlife in the United States (MIDUS 1), 1995-1996. Inter-university Consortium for Political and Social Research. 2020. 10.3886/ICPSR02760.v19

[14] Ryff CD, Almeida DM, Ayanian JZ, Carr DS, Cleary PD, Coe C, Davidson RJ, Krueger RF, Lachman ME, Marks NF, Mroczek DK, Seeman TE, Seltzer MM, et al. Midlife in the United States (MIDUS 2), 2004-2006. Inter-university Consortium for Political and Social Research. 2021. 10.3886/ICPSR04652.v8

[15] Blanchflower DG, Oswald AJ. Is well-being U-shaped over the life cycle? Soc Sci Med. 2008; 66:1733–49. 10.1016/j.socscimed.2008.01.030 18316146

[16] Blanchflower DG. Is happiness U-shaped everywhere? Age and subjective well-being in 145 countries. J Popul Econ. 2021; 34:575–624. 10.1007/s00148-020-00797-z 32929308 PMC7480662

[17] Lim HJ, Min DK, Thorpe L, Lee CH. Trajectories of Life Satisfaction and their Predictors among Korean Older Adults. BMC Geriatr. 2017; 17:89. 10.1186/s12877-017-0485-5 28420335 PMC5395837

[18] Costa PT Jr, Zonderman AB, McCrae RR, Cornoni-Huntley J, Locke BZ, Barbano HE. Longitudinal analyses of psychological well-being in a national sample: stability of mean levels. J Gerontol. 1987; 42:50–5. 10.1093/geronj/42.1.50 3794196

[19] Lucas RE. Adaptation and the Set-Point Model of Subjective Well-Being: Does Happiness Change After Major Life Events? Curr Dir Psychol Sci. 2007; 16:75–9. 10.1111/j.1467-8721.2007.00479.x

[20] Diener E, Lucas RE, Scollon CN. Beyond the hedonic treadmill: revising the adaptation theory of well-being. Am Psychol. 2006; 61:305–14. 10.1037/0003-066X.61.4.305 16719675

[21] Brockman J, Etcoff N. What Are You Optimistic About?: Today’s Leading Thinkers on Why Things Are Good and Getting Better. Simon & Schuster Ltd. 2007. 169–72.

[22] Mobile Health (mHealth) App Market - Global Outlook to 2026: Johnson and Johnson, Omron, Airstrip, Philips, Qualcomm Dominate. 2021. Research and Markets. https://www.prnewswire.com/news-releases/mobile-health-mhealth-app-market---global-outlook-to-2026-johnson-and-johnson-omron-airstrip-philips-qualcomm-dominate-301451438.html

[23] Webb CA, Rosso IM, Rauch SL. Internet-Based Cognitive-Behavioral Therapy for Depression: Current Progress and Future Directions. Harv Rev Psychiatry. 2017; 25:114–22. 10.1097/HRP.0000000000000139 28475503 PMC5421393

[24] Richards D, Richardson T. Computer-based psychological treatments for depression: a systematic review and meta-analysis. Clin Psychol Rev. 2012; 32:329–42. 10.1016/j.cpr.2012.02.004 22466510

[25] van Straten A, Cuijpers P, Smits N. Effectiveness of a web-based self-help intervention for symptoms of depression, anxiety, and stress: randomized controlled trial. J Med Internet Res. 2008; 10:e7. 10.2196/jmir.954 18364344 PMC2483843

[26] Jernelöv S, Lekander M, Blom K, Rydh S, Ljótsson B, Axelsson J, Kaldo V. Efficacy of a behavioral self-help treatment with or without therapist guidance for co-morbid and primary insomnia--a randomized controlled trial. BMC Psychiatry. 2012; 12:5. 10.1186/1471-244X-12-5 22264332 PMC3328261

[27] Hoek W, Schuurmans J, Koot HM, Cuijpers P. Effects of Internet-based guided self-help problem-solving therapy for adolescents with depression and anxiety: a randomized controlled trial. PLoS One. 2012; 7:e43485. 10.1371/journal.pone.0043485 22952691 PMC3432036

[28] Zhavoronkov A, Ivanenkov YA, Aliper A, Veselov MS, Aladinskiy VA, Aladinskaya AV, Terentiev VA, Polykovskiy DA, Kuznetsov MD, Asadulaev A, Volkov Y, Zholus A, Shayakhmetov RR, et al. Deep learning enables rapid identification of potent DDR1 kinase inhibitors. Nat Biotechnol. 2019; 37:1038–40. 10.1038/s41587-019-0224-x 31477924

[29] Bhatt A. Artificial intelligence in managing clinical trial design and conduct: Man and machine still on the learning curve? Perspect Clin Res. 2021; 12:1–3. 10.4103/picr.PICR_312_20 33816201 PMC8011519

[30] Rondina JM, Filippone M, Girolami M, Ward NS. Decoding post-stroke motor function from structural brain imaging. Neuroimage Clin. 2016; 12:372–80. 10.1016/j.nicl.2016.07.014 27595065 PMC4995603

[31] Martin-Isla C, Campello VM, Izquierdo C, Raisi-Estabragh Z, Baeßler B, Petersen SE, Lekadir K. Image-Based Cardiac Diagnosis With Machine Learning: A Review. Front Cardiovasc Med. 2020; 7:1. 10.3389/fcvm.2020.00001 32039241 PMC6992607

[32] Patel B, Makaryus AN. Artificial Intelligence Advances in the World of Cardiovascular Imaging. Healthcare (Basel). 2022; 10:154. 10.3390/healthcare10010154 35455912 PMC9030845

[33] Jiang F, Jiang Y, Zhi H, Dong Y, Li H, Ma S, Wang Y, Dong Q, Shen H, Wang Y. Artificial intelligence in healthcare: past, present and future. Stroke Vasc Neurol. 2017; 2:230–43. 10.1136/svn-2017-000101 29507784 PMC5829945

[34] Horvath S. DNA methylation age of human tissues and cell types. Genome Biol. 2013; 14:R115. 10.1186/gb-2013-14-10-r115 24138928 PMC4015143

[35] Galkin F, Mamoshina P, Aliper A, de Magalhães JP, Gladyshev VN, Zhavoronkov A. Biohorology and biomarkers of aging: Current state-of-the-art, challenges and opportunities. Ageing Res Rev. 2020; 60:101050. 10.1016/j.arr.2020.101050 32272169

[36] Bobrov E, Georgievskaya A, Kiselev K, Sevastopolsky A, Zhavoronkov A, Gurov S, Rudakov K, Del Pilar Bonilla Tobar M, Jaspers S, Clemann S. PhotoAgeClock: deep learning algorithms for development of non-invasive visual biomarkers of aging. Aging (Albany NY). 2018; 10:3249–59. 10.18632/aging.101629 30414596 PMC6286834

[37] Mamoshina P, Volosnikova M, Ozerov IV, Putin E, Skibina E, Cortese F, Zhavoronkov A. Machine Learning on Human Muscle Transcriptomic Data for Biomarker Discovery and Tissue-Specific Drug Target Identification. Front Genet. 2018; 9:242. 10.3389/fgene.2018.00242 30050560 PMC6052089

[38] Putin E, Mamoshina P, Aliper A, Korzinkin M, Moskalev A, Kolosov A, Ostrovskiy A, Cantor C, Vijg J, Zhavoronkov A. Deep biomarkers of human aging: Application of deep neural networks to biomarker development. Aging (Albany NY). 2016; 8:1021–33. 10.18632/aging.100968 27191382 PMC4931851

[39] Zhavoronkov A, Mamoshina P, Vanhaelen Q, Scheibye-Knudsen M, Moskalev A, Aliper A. Artificial intelligence for aging and longevity research: Recent advances and perspectives. Ageing Res Rev. 2019; 49:49–66. 10.1016/j.arr.2018.11.003 30472217

[40] Mamoshina P, Kochetov K, Cortese F, Kovalchuk A, Aliper A, Putin E, Scheibye-Knudsen M, Cantor CR, Skjodt NM, Kovalchuk O, Zhavoronkov A. Blood Biochemistry Analysis to Detect Smoking Status and Quantify Accelerated Aging in Smokers. Sci Rep. 2019; 9:142. 10.1038/s41598-018-35704-w 30644411 PMC6333803

[41] Zhavoronkov A, Mamoshina P. Deep Aging Clocks: The Emergence of AI-Based Biomarkers of Aging and Longevity. Trends Pharmacol Sci. 2019; 40:546–9. 10.1016/j.tips.2019.05.004 31279569

[42] Galkin F, Mamoshina P, Kochetov K, Sidorenko D, Zhavoronkov A. DeepMAge: A Methylation Aging Clock Developed with Deep Learning. Aging Dis. 2021; 12:1252–62. 10.14336/AD.2020.1202 34341706 PMC8279523

[43] Galkin F, Mamoshina P, Aliper A, Putin E, Moskalev V, Gladyshev VN, Zhavoronkov A. Human Gut Microbiome Aging Clock Based on Taxonomic Profiling and Deep Learning. iScience. 2020; 23:101199. 10.1016/j.isci.2020.101199 32534441 PMC7298543

[44] Levine ME, Lu AT, Quach A, Chen BH, Assimes TL, Bandinelli S, Hou L, Baccarelli AA, Stewart JD, Li Y, Whitsel EA, Wilson JG, Reiner AP, et al. An epigenetic biomarker of aging for lifespan and healthspan. Aging (Albany NY). 2018; 10:573–91. 10.18632/aging.101414 29676998 PMC5940111

[45] Liu Z, Kuo PL, Horvath S, Crimmins E, Ferrucci L, Levine M. A new aging measure captures morbidity and mortality risk across diverse subpopulations from NHANES IV: A cohort study. PLoS Med. 2018; 15:e1002718. 10.1371/journal.pmed.1002718 30596641 PMC6312200

[46] Lu AT, Quach A, Wilson JG, Reiner AP, Aviv A, Raj K, Hou L, Baccarelli AA, Li Y, Stewart JD, Whitsel EA, Assimes TL, Ferrucci L, Horvath S. DNA methylation GrimAge strongly predicts lifespan and healthspan. Aging (Albany NY). 2019; 11:303–27. 10.18632/aging.101684 30669119 PMC6366976

[47] Mamoshina P, Zhavoronkov A. Deep Integrated Biomarkers of Aging. Biomarkers of Human Aging. Healthy Ageing and Longevity. 2019; 10:281–91. 10.1007/978-3-030-24970-0_18

[48] Mamoshina P, Vieira A, Putin E, Zhavoronkov A. Applications of Deep Learning in Biomedicine. Mol Pharm. 2016; 13:1445–54. 10.1021/acs.molpharmaceut.5b00982 27007977

[49] Aliper AM, Galkin F, Zavoronkovs A. Aging markers of human microbiome and microbiomic aging clock. US Patent Application Publication. 2020: US20200075127A1. https://patents.google.com/patent/US20200075127A1/en

[50] Aliper AM, Zavoronkovs A, Ozerov I, Bozdaganyan ME, Artemov AV. Method of treating senescence with multi-stage longevity therapeutics. United States Patent. 2022: US11260078B2. https://patents.google.com/patent/US11260078B2/en

[51] Aliper A, Putin E, Zavoronkovs A. Deep transcriptomic markers of human biological aging and methods of determining a biological aging clock. United States Patent Application Publication. 2018: US20190034581A1. https://patents.google.com/patent/US20190034581A1/en

[52] Horvath S. Method to estimate the age of tissues and cell types based on epigenetic markers. European Patent Specification. 2021: EP3049535B1. https://patents.google.com/patent/EP3049535B1/en

[53] Zhang K, Hannum G, Ideker T, Friend SH, Guinney J. Methods for predicting age and identifying agents that induce or inhibit premature aging. United States Patent Application Publication. 2015: US20150259742A1. https://patents.google.com/patent/US20150259742A1/en

[54] Galkin F, Kochetov KS, Mamoshina P, Zavoronkovs A. Methylation data signatures of aging and methods of determining a methylation aging clock. United States Patent Application Publication. 2022: US20220005552A1. https://patents.google.com/patent/US20220005552A1/en

[55] Zhavoronkov A. Geroprotective and senoremediative strategies to reduce the comorbidity, infection rates, severity, and lethality in gerophilic and gerolavic infections. Aging (Albany NY). 2020; 12:6492–510. 10.18632/aging.102988 32229705 PMC7202545

[56] Galkin F, Parish A, Bischof E, Zhang J, Mamoshina P, Zhavoronkov A. Increased Pace of Aging in COVID-Related Mortality. Life (Basel). 2021; 11:730. 10.3390/life11080730 34440474 PMC8401657

[57] Zhavoronkov A. Longevity expectations in the pension fund, insurance, and employee benefits industries. Psychol Res Behav Manag. 2015; 8:27–39. 10.2147/PRBM.S75440 25653568 PMC4309776

[58] McCrory C, Fiorito G, Hernandez B, Polidoro S, O’Halloran AM, Hever A, Ni Cheallaigh C, Lu AT, Horvath S, Vineis P, Kenny RA. GrimAge Outperforms Other Epigenetic Clocks in the Prediction of Age-Related Clinical Phenotypes and All-Cause Mortality. J Gerontol A Biol Sci Med Sci. 2021; 76:741–9. 10.1093/gerona/glaa286 33211845 PMC8087266

[59] Mamoshina P, Kochetov K, Putin E, Cortese F, Aliper A, Lee WS, Ahn SM, Uhn L, Skjodt N, Kovalchuk O, Scheibye-Knudsen M, Zhavoronkov A. Population Specific Biomarkers of Human Aging: A Big Data Study Using South Korean, Canadian, and Eastern European Patient Populations. J Gerontol A Biol Sci Med Sci. 2018; 73:1482–90. 10.1093/gerona/gly005 29340580 PMC6175034

[60] Belsky DW, Caspi A, Arseneault L, Baccarelli A, Corcoran DL, Gao X, Hannon E, Harrington HL, Rasmussen LJ, Houts R, Huffman K, Kraus WE, Kwon D, et al. Quantification of the pace of biological aging in humans through a blood test, the DunedinPoAm DNA methylation algorithm. Elife. 2020; 9:e54870. 10.7554/eLife.54870 32367804 PMC7282814

[61] Boks MP, van Mierlo HC, Rutten BP, Radstake TR, De Witte L, Geuze E, Horvath S, Schalkwyk LC, Vinkers CH, Broen JC, Vermetten E. Longitudinal changes of telomere length and epigenetic age related to traumatic stress and post-traumatic stress disorder. Psychoneuroendocrinology. 2015; 51:506–12. 10.1016/j.psyneuen.2014.07.011 25129579

[62] Brody GH, Yu T, Chen E, Beach SR, Miller GE. Family-centered prevention ameliorates the longitudinal association between risky family processes and epigenetic aging. J Child Psychol Psychiatry. 2016; 57:566–74. 10.1111/jcpp.12495 26680699 PMC4836970

[63] Jovanovic T, Vance LA, Cross D, Knight AK, Kilaru V, Michopoulos V, Klengel T, Smith AK. Exposure to Violence Accelerates Epigenetic Aging in Children. Sci Rep. 2017; 7:8962. 10.1038/s41598-017-09235-9 28827677 PMC5566406

[64] Anderson JA, Johnston RA, Lea AJ, Campos FA, Voyles TN, Akinyi MY, Alberts SC, Archie EA, Tung J. High social status males experience accelerated epigenetic aging in wild baboons. Elife. 2021; 10:e66128. 10.7554/eLife.66128 33821798 PMC8087445

[65] Zannas AS, Arloth J, Carrillo-Roa T, Iurato S, Röh S, Ressler KJ, Nemeroff CB, Smith AK, Bradley B, Heim C, Menke A, Lange JF, Brückl T, et al. Lifetime stress accelerates epigenetic aging in an urban, African American cohort: relevance of glucocorticoid signaling. Genome Biol. 2015; 16:266. 10.1186/s13059-015-0828-5 26673150 PMC4699359

[66] Gesquiere LR, Learn NH, Simao MC, Onyango PO, Alberts SC, Altmann J. Life at the top: rank and stress in wild male baboons. Science. 2011; 333:357–60. 10.1126/science.1207120 21764751 PMC3433837

[67] Mitina M, Young S, Zhavoronkov A. Psychological aging, depression, and well-being. Aging (Albany NY). 2020; 12:18765–77. 10.18632/aging.103880 32950973 PMC7585090

[68] Zhavoronkov A, Kochetov K, Diamandis P, Mitina M. PsychoAge and SubjAge: development of deep markers of psychological and subjective age using artificial intelligence. Aging (Albany NY). 2020; 12:23548–77. 10.18632/aging.202344 33303702 PMC7762465

[69] Carstensen LL. Socioemotional Selectivity Theory: The Role of Perceived Endings in Human Motivation. Gerontologist. 2021; 61:1188–96. 10.1093/geront/gnab116 34718558 PMC8599276

[70] Fung HH, Chu ST, Jiang D, Chen AX, Ng CC. Contrasting the Effects of Mortality Salience and Future Time Limitation on Goal Prioritization in Older and Younger Adults. J Gerontol B Psychol Sci Soc Sci. 2020; 75:2112–21. 10.1093/geronb/gbz133 31628456

[71] Fung HH, Lai P, Ng R. Age differences in social preferences among Taiwanese and Mainland Chinese: the role of perceived time. Psychol Aging. 2001; 16:351–6. 10.1037//0882-7974.16.2.351 11405322

[72] Fung HH, Carstensen LL. Goals change when life’s fragility is primed: Lessons learned from older adults, the September 11 attacks and sars. Soc Cogn. 2006; 24:248–78. 10.1521/soco.2006.24.3.248

[73] Jacobs JM, Maaravi Y, Stessman J. Optimism and Longevity Beyond Age 85. J Gerontol A Biol Sci Med Sci. 2021; 76:1806–13. 10.1093/gerona/glab051 33609364

[74] Fekihal MA. Self-Organizing Map Approach for Identifying Mental Disorders. Int J Comput Appl. 2012; 45:6.

[75] Tervonen J, Puttonen S, Sillanpää MJ, Hopsu L, Homorodi Z, Keränen J, Pajukanta J, Tolonen A, Lämsä A, Mäntyjärvi J. Personalized mental stress detection with self-organizing map: From laboratory to the field. Comput Biol Med. 2020; 124:103935. 10.1016/j.compbiomed.2020.103935 32771674

[76] Albert PR. Why is depression more prevalent in women? J Psychiatry Neurosci. 2015; 40:219–21. 10.1503/jpn.150205 26107348 PMC4478054

[77] Datta Gupta N, Etcoff NL, Jaeger MM. Beauty in Mind: The Effects of Physical Attractiveness on Psychological Well-Being and Distress. J Happiness Stud. 2016; 17:1313–25. 10.1007/s10902-015-9644-6

[78] Snyder M, Tanke ED, Berscheid E. Social perception and interpersonal behavior: On the self-fulfilling nature of social stereotypes. J Pers Soc Psychol. 1977; 35:656–66. 10.1037/0022-3514.35.9.656

[79] Sharpee TO, Destexhe A, Kawato M, Sekulić V, Skinner FK, Wójcik DK, Chintaluri C, Cserpán D, Somogyvári Z, Kim JK, Kilpatrick ZP, Bennett MR, Josić K, et al. 25th Annual Computational Neuroscience Meeting: CNS-2016. BMC Neurosci. 2016; 17 (Suppl 1):54. 10.1186/s12868-016-0283-6 27534393 PMC5001212

[80] von Gunten A, Mosimann UP, Antonietti JP. A longitudinal study on delirium in nursing homes. Am J Geriatr Psychiatry. 2013; 21:963–72. 10.1016/j.jagp.2013.01.003 23567403

[81] Paulus MP, Yu AJ. Emotion and decision-making: affect-driven belief systems in anxiety and depression. Trends Cogn Sci. 2012; 16:476–83. 10.1016/j.tics.2012.07.009 22898207 PMC3446252

[82] Foster ED, Deardorff A. Open Science Framework (OSF). J Med Libr Assoc. 2017; 105:203–6. 10.5195/jmla.2017.88

[83] Kursa MB, Rudnicki WR. Feature Selection with the Boruta Package. J Stat Softw. 2010; 36:1–13. 10.18637/jss.v036.i11

[84] Maas AL, Hannun AY, Ng AY. Rectifier nonlinearities improve neural network acoustic models. ICML Workshop on Deep Learning for Audio, Speech and Language Processing. 2013. https://citeseerx.ist.psu.edu/viewdoc/download?doi=10.1.1.693.1422&rep=rep1&type=pdf

[85] Srivastava N, Hinton G, Krizhevsky A, Sutskever I, Salakhutdinov R. Dropout: a simple way to prevent neural networks from overfitting. J Mach Learn Res. 2014; 15:1929–58.

[86] Kohonen T. The self-organizing map. Proceedings of the IEEE. 1990; 78:1464–80. 10.1109/5.58325

