# Supplementary material for: Optimizing future well-being with artificial intelligence: self-organizing maps (SOMs) for the identification of islands of emotional stability
Source: Aging (Albany NY). 2022 Jun 20;14(12):4935–58. doi: 10.18632/aging.204061 (PMC9271294; doi:10.18632/aging.204061)
Supplement: Supplementary Figures [file aging-14-204061-s001.pdf]

SUPPLEMENTARY FIGURES

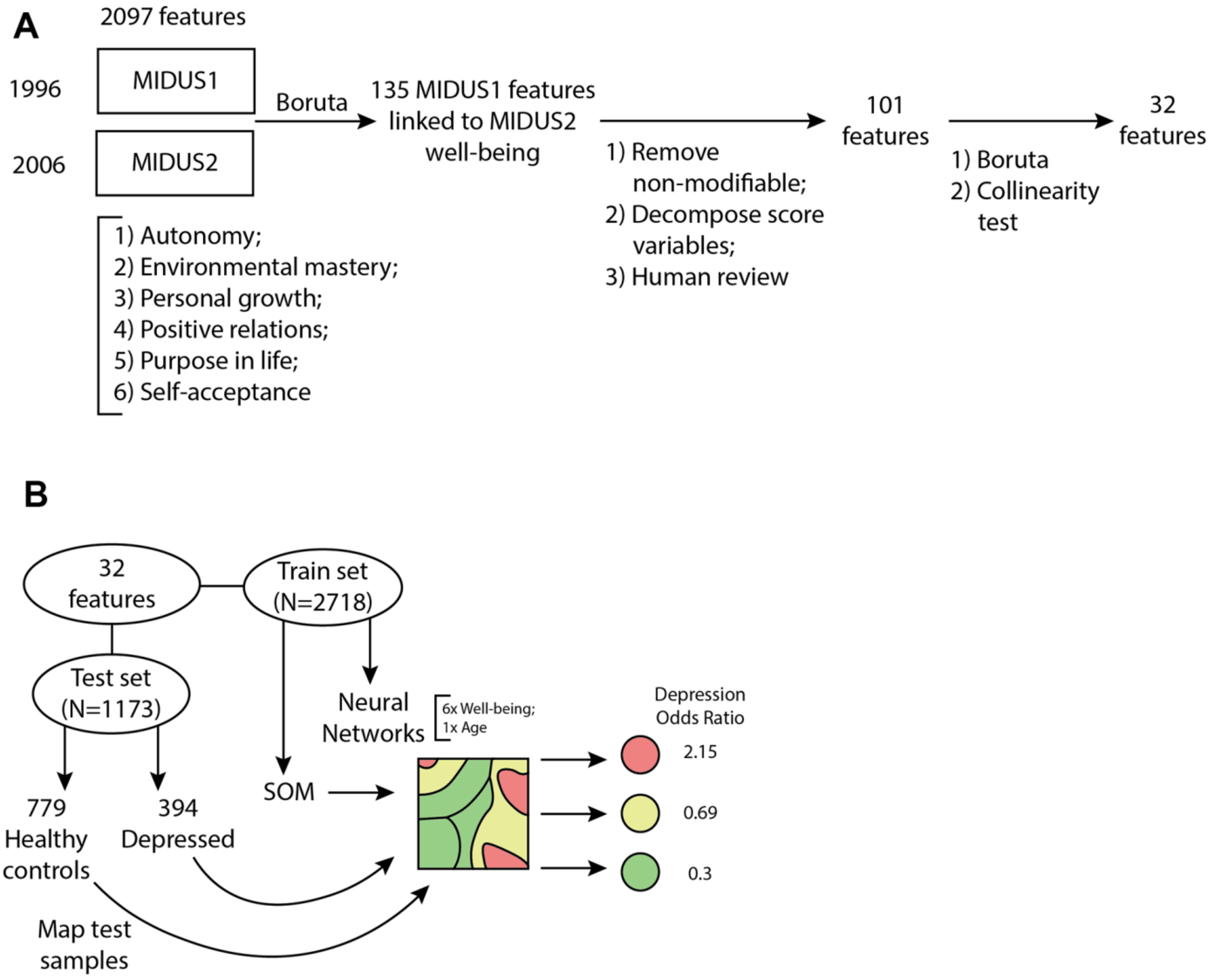

Supplementary Figure 1. The flow of the research presented in this study. (**A**) Feature selection procedure. A total of 2097 variables present in both MIDUS1 and MIDUS2 US-wide survey were considered. Using Boruta, a method of feature selection, we selected only the variables whose values in MIDUS1 were representative of the six Ryff scale well-being parameters in MIDUS2. The initial list of 135 variables was reduced down to 32 features by reapplying Boruta, removing non-modifiable and strongly correlated features. After the first round of Boruta filtering, some selected features represented linear scores. Such scores were decomposed into their elemental variables before the second round of feature reduction. (**B**) Data partition and models built. Only participants assessed in both MIDUS waves were considered. This cohort was divided into the training set (N=2718) containing only non-depressed individuals and the test set. The same training set was used to train both the aging clock and the SOM. The test set was further subdivided into the cohort of non-depressed (N=779) and the depressed (N=394) people, as described in MIDUS1. The SOM was hierarchically clustered prior to mapping the participants from the test set. The independently defined clusters, however, display regions with an increased presence of depressed people and people with low psychological well-being.

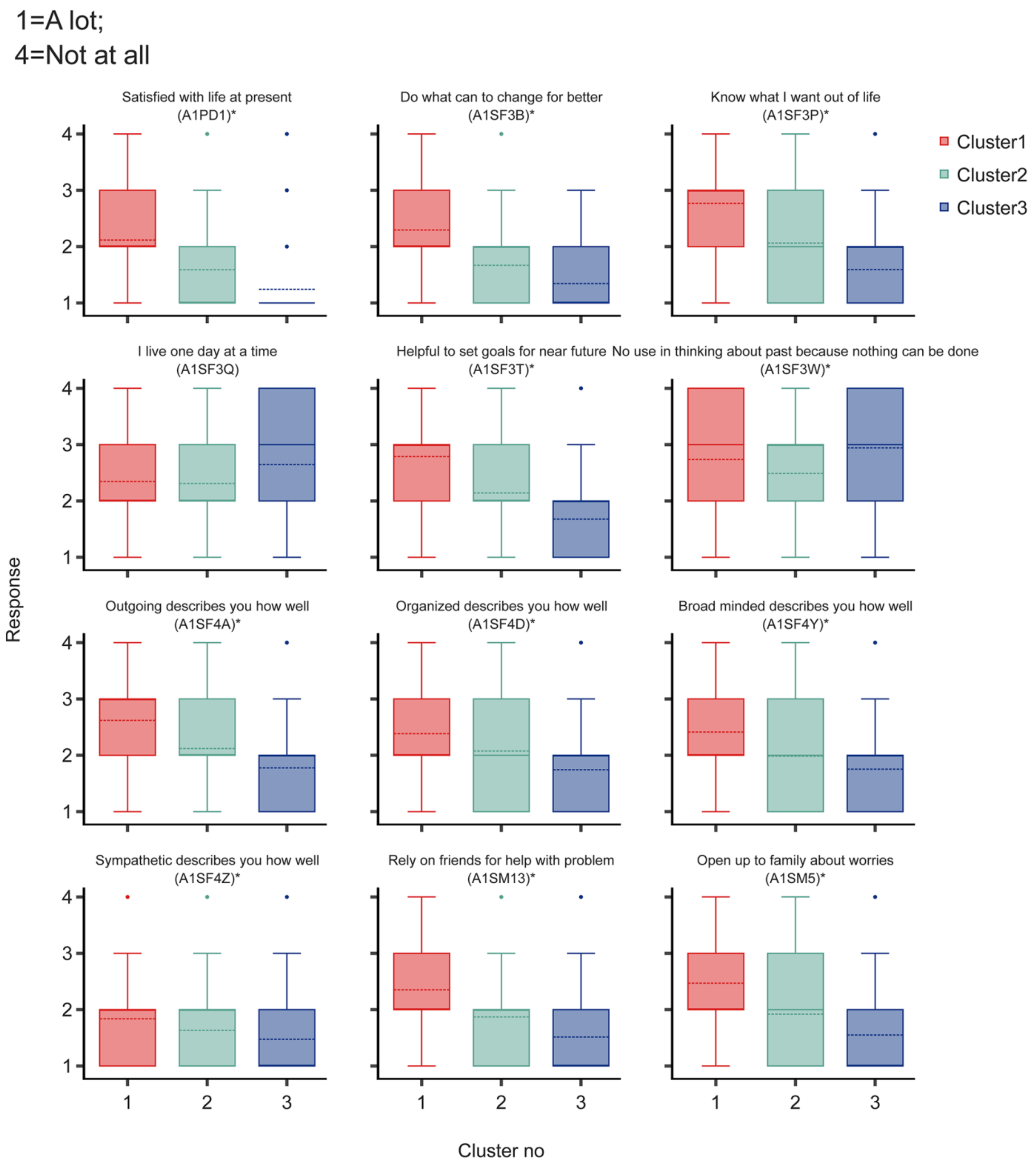

Supplementary Figure 2. Feature distribution across the three clusters identified within the SOM. Only MIDUS features with a four-point scale are shown. Asterisks mark the variables with significant ($p < 0.01$, U-test) differences in mean feature value between cluster-1 (depression odds = 2.15) and cluster-2 (depression odds = 0.69). Boxes represent the interquartile region, whiskers protrude no further than 1.5 times the IQR, solid horizontal lines represent the median, and dotted lines represent the mean.

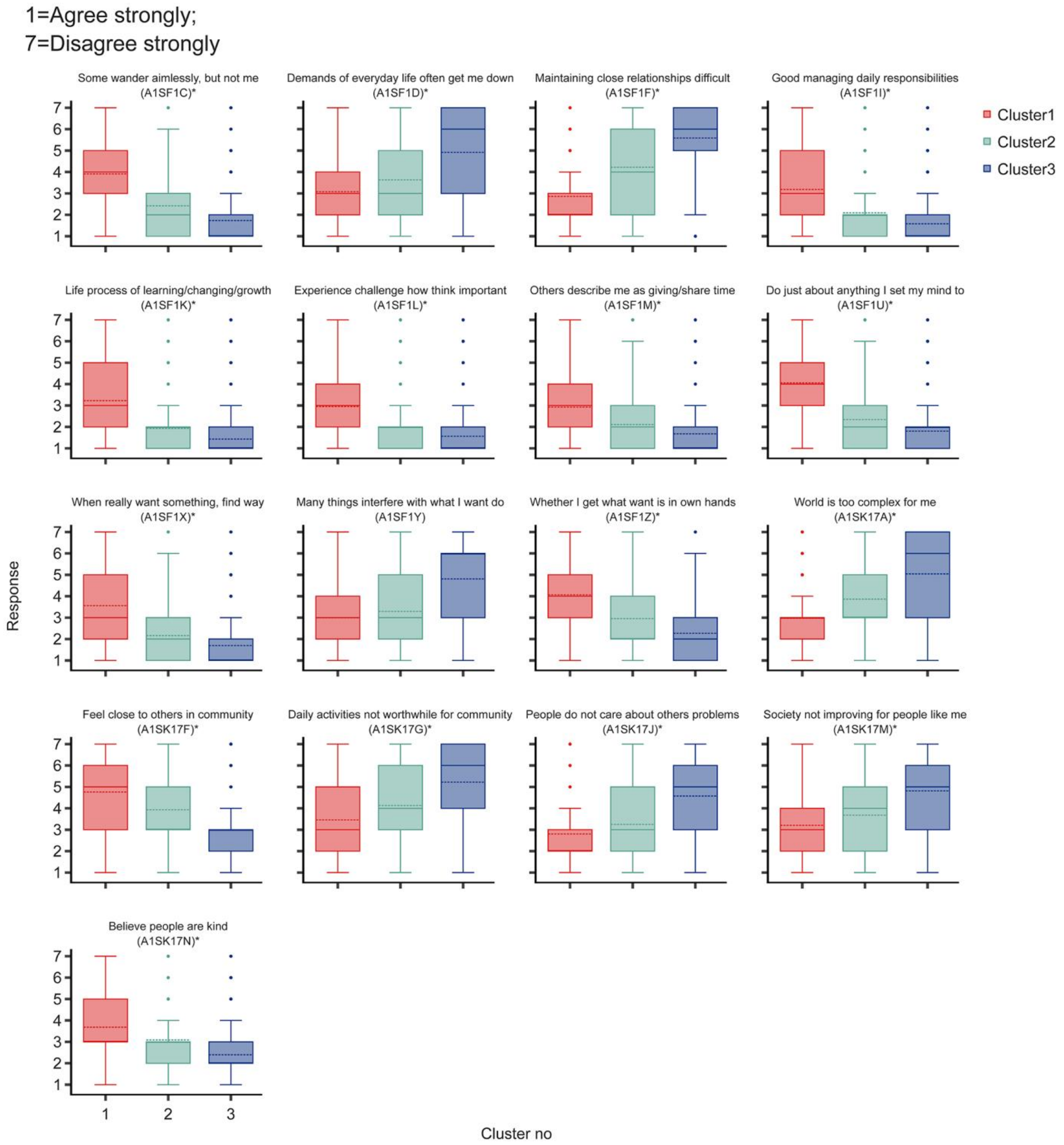

Supplementary Figure 3. Feature distribution across the three clusters identified within the SOM. Only MIDUS features with a seven-point scale are shown. Asterisks mark the variables with significant (p <0.01, U-test) differences in mean feature value between cluster -1 (depression odds = 2.15) and cluster-2 (depression odds = 0.69). Boxes represent the interquartile region, whiskers protrude no further than 1.5 times the IQR, solid horizontal lines represent the median, and dotted lines represent the mean.

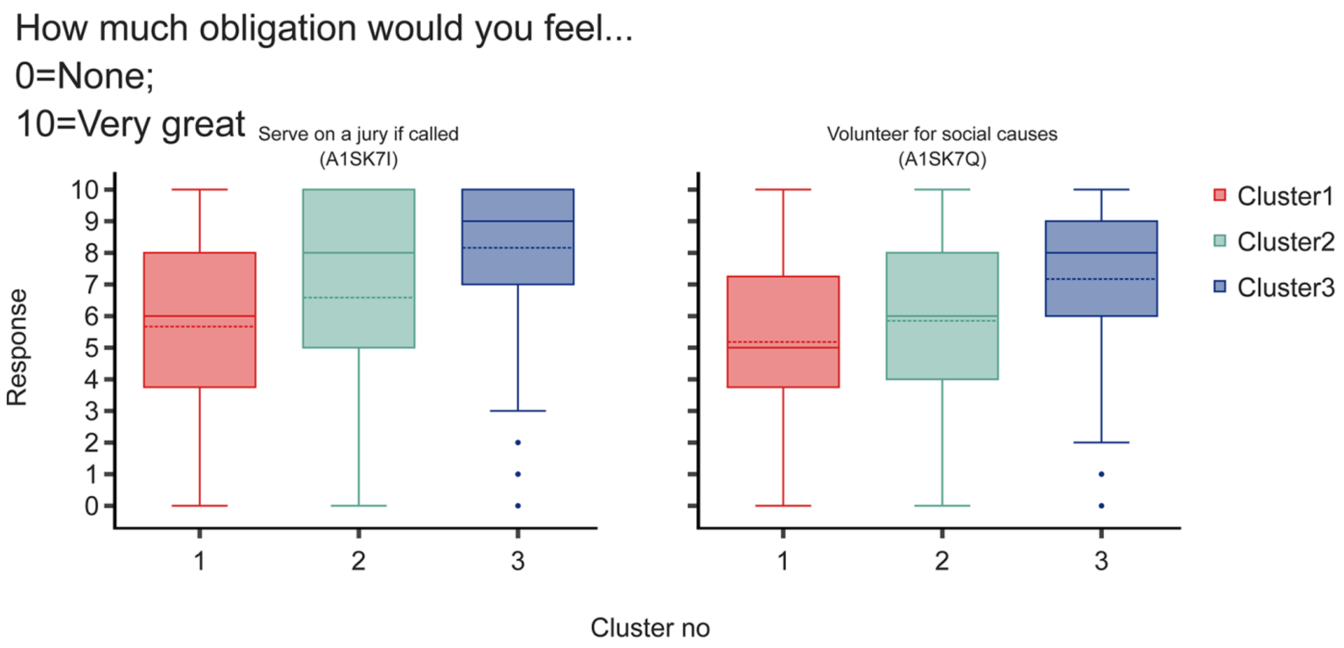

Supplementary Figure 4. Feature distribution across the three clusters identified within the SOM. Only MIDUS features with a ten-point scale are shown. Asterisks mark the variables with significant (p <0.01, U-test) differences in mean feature value between cluster -1 (depression odds = 2.15) and cluster-2 (depression odds = 0.69). Boxes represent the interquartile region, whiskers protrude no further than 1.5 times the IQR, solid horizontal lines represent the median, and dotted lines represent the mean.

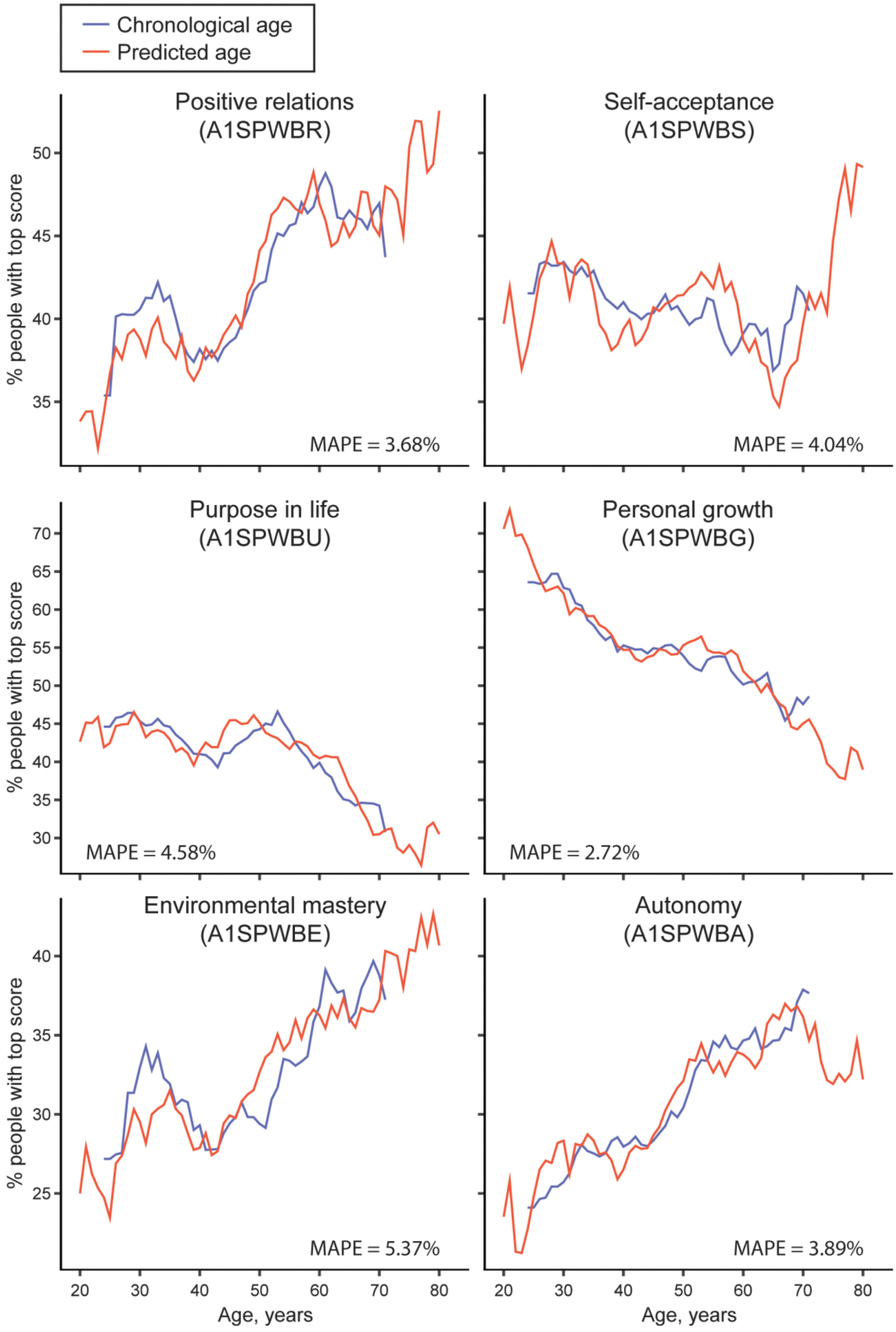

Supplementary Figure 5. The deep learning model replicates age-associated changes in different well-being aspects. "Top score" is defined as 19–21 points out of 21 for each well-being aspect. MAPE is the mean absolute percentage error between the predicted and chronological age lines. All plots are obtained from the training set (N = 2718), and predicted age values were obtained in five-fold cross-validation.
