# Supplementary material for: Optimizing future well-being with artificial intelligence: self-organizing maps (SOMs) for the identification of islands of emotional stability
Source: Aging (Albany NY). 2022 Jun 20;14(12):4935–58. doi: 10.18632/aging.204061 (PMC9271294; doi:10.18632/aging.204061)
Supplement: Supplementary Tables [file aging-14-204061-s002.pdf]

SUPPLEMENTARY TABLES

Supplementary Table 1. Predictor accuracy metrics for all target variables.

Target variable	Variable description	MAPE, %		MAE, units		Pearson's r	
		Training	Test	Training	Test	Training	Test
A1PAGE_M2(adjusted)		9.50	9.79	4.29	4.30	0.91	0.92
A1PAGE_M2	Current age	20.57	21.39	8.97	9.06	0.46	0.47
A1PAGE_M2(baseline)		23.79	23.31	10.31	10.32	0.00	0.00
B1SPWBA1	Autonomy in 10 years	15.84	15.81	2.36	2.36	0.35	0.34
B1SPWBE1	Environmental mastery in 10 years	14.38	18.51	2.10	2.28	0.51	0.48
B1SPWBG1	Personal growth in 10 years	13.91	15.97	2.08	2.25	0.52	0.53
B1SPWBR1	Positive relations in 10 years	16.52	18.47	2.31	2.46	0.58	0.61
B1SPWBS1	Self-acceptance in 10 years	18.42	22.53	2.39	2.62	0.54	0.53
B1SPWBU1	Purpose in life in 10 years	17.25	19.27	2.42	2.59	0.43	0.39

Training metrics were obtained in five-fold cross-validation. MAE is measured in years for age and in points for well-being. The baseline model for age prediction was the median age assignment.

Supplementary Table 2. Relative importance analysis shows that future predicted well-being is more closely associated with actual future well-being than current well-being, age, or sex.

MIDUS2 target variable	Target description	Is male	Chronological age	Predicted MIDUS2 variable	MIDUS1 actual well-being variables					
					A1SPWBR	A1SPWBS	A1SPWBA	A1SPWBG	A1SPWBE	A1SPWBU
B1SPWBR1	Close relations	-0.26 *	0.02	0.43 *	0.23 *	0.08	-0.02	0.02	0.01	0.05
B1SPWBS1	Self-acceptance	0.00	0.01	0.52 *	0.02	0.38 *	0.00	0.00	-0.01	-0.01
B1SPWBA1	Autonomy	0.15 *	0.01	0.47 *	0.01	0.00	0.35 *	0.00	0.00	0.01
B1SPWBG1	Personal growth	-0.32 *	-0.01	0.55 *	0.01	0.07	0.00	0.19 *	-0.02	0.04
B1SPWBE1	Environmental mastery	0.25 *	0.02	0.63 *	0.01	0.09	0.00	0.01	0.10 *	0.00
B1SPWBU1	Purpose in life	0.00	-0.03	0.39 *	0.00	0.00	0.01	0.09	0.02	0.28 *

EN linear regressions were trained to approximate each MIDUS2 well-being variable using sex (binary “Is male”), age, predicted counterpart of the MIDUS2 variable, and all six MIDUS1 well-being variables. The largest coefficients in all cases belonged to model predictions, attesting to them being more important determinants of future well-being than current well-being. Asterisks mark the EN coefficients larger than 0.1 in absolute terms. EN is elastic net (see Methods).

Supplementary Table 3. Correlation between mean well-being scores and depression odds for people mapped to the same SOM cells.

Time point	Well-being parameter	Pearson's r
MIDUS1	Close relationships	-0.24*
	Self-acceptance	-0.36*
	Autonomy	-0.24*
	Personal growth	-0.16*
	Environmental mastery	-0.33
	Purpose in life	-0.15*
MIDUS2	Close relationships	-0.21*
	Self-acceptance	-0.25*
	Autonomy	-0.12*
	Personal growth	-0.11
	Environmental mastery	-0.25*
	Purpose in life	-0.06

"*" marks the coefficients with $p < 0.01$.
